# Biomaterials in Drug Delivery: Advancements in Cancer and Diverse Therapies—Review

**DOI:** 10.3390/ijms25063126

**Published:** 2024-03-08

**Authors:** Anna Drabczyk, Sonia Kudłacik-Kramarczyk, Mateusz Jamroży, Marcel Krzan

**Affiliations:** 1 CBRTP SA—Research and Development Center of Technology for Industry, 3A Ludwika Waryńskiego St., 00-645 Warsaw, Poland; 2 Jerzy Haber Institute of Catalysis and Surface Chemistry, Polish Academy of Sciences, 8 Niezapominajek St., 30-239 Krakow, Poland; mateusz.jamrozy@student.pk.edu.pl (M.J.); marcel.krzan@ikifp.edu.pl (M.K.); 3 Department of Materials Engineering, Faculty of Materials Engineering and Physics, Cracow University of Technology, 37 Jana Pawła II Av., 31-864 Krakow, Poland

**Keywords:** nanomaterials, biomaterials, drug carriers, nano drug delivery, DNA, fibrin, pectin, hyaluronic acid, chitosan, fibroin, anti-cancer therapy

## Abstract

Nano-sized biomaterials are innovative drug carriers with nanometric dimensions. Designed with biocompatibility in mind, they enable precise drug delivery while minimizing side effects. Controlled release of therapeutic substances enhances efficacy, opening new possibilities for treating neurological and oncological diseases. Integrated diagnostic-therapeutic nanosystems allow real-time monitoring of treatment effectiveness, which is crucial for therapy personalization. Utilizing biomaterials as nano-sized carriers in conjunction with drugs represents a promising direction that could revolutionize the field of pharmaceutical therapy. Such carriers represent groundbreaking drug delivery systems on a nanometric scale, designed with biocompatibility in mind, enabling precise drug delivery while minimizing side effects. Using biomaterials in synergy with drugs demonstrates significant potential for a revolutionary impact on pharmaceutical therapy. Conclusions drawn from the review indicate that nano-sized biomaterials constitute an innovative tool that can significantly improve therapy effectiveness and safety, especially in treating neurological and oncological diseases. These findings should guide researchers towards further studies to refine nano-sized biomaterials, assess their effectiveness under various pathological conditions, and explore diagnostic-therapeutic applications. Ultimately, these results underscore the promising nature of nano-sized biomaterials as advanced drug carriers, ushering in a new era in nanomedical therapy.

## 1. Introduction

Nanomaterials, as drug carriers, occupy a central position in the field of applied nanotechnology in medicine [1]. These advanced materials, with nanometric dimensions, exhibit unique properties that make them effective carriers for therapeutic substances [2]. Nanomaterials can be designed with biocompatibility in mind, minimizing immunological reactions and the risk of toxicity [3]. Their structure and properties can be tailored to specific therapeutic requirements, enabling precise drug delivery to particular cells or tissues [4].

Moreover, nanomaterials offer controlled drug release, a critical factor in enhancing therapeutic efficacy [5]. This control can be achieved through chemical or physical modifications of the nanobiomaterial structure, allowing the concentrated release of therapeutic substances at specific locations and times [6].

**Figure 1:**
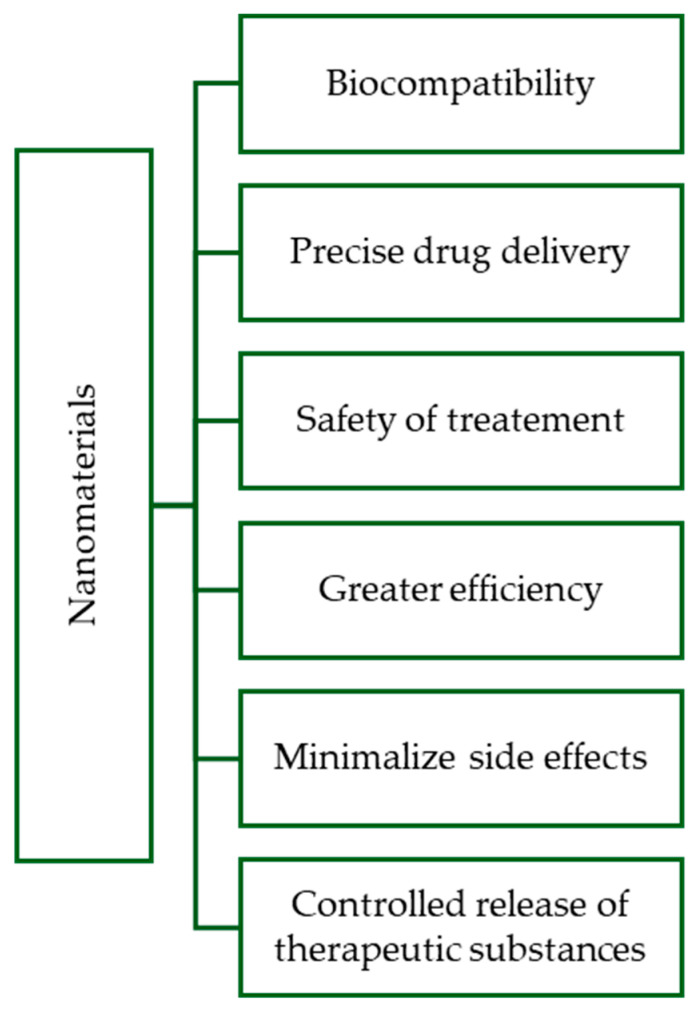
Properties of nanomaterials that give them an advantage over other materials in delivering therapeutic substances.

Nanomaterials used as drug carriers also possess the ability to penetrate biological barriers, such as the blood-brain barrier or vascular barrier, enabling targeted drug delivery to challenging areas of the body. This opens new possibilities in treating neurological or oncological diseases [7]. Finally, nanomaterials serve as platforms for advanced diagnostic methods, allowing real-time monitoring of treatment processes [8]. Integrated diagnostic-therapeutic nanosystems (theranostics) enable real-time monitoring of treatment effectiveness [9]. The figure below (Figure 1) presents the basic properties of nanomaterials that make them so readily used in drug delivery therapies.

Due to their unique physicochemical properties, nanomaterials represent a promising field in drug delivery. Compared to macro-molecules or other nanostructures, nanomaterials exhibit specific characteristics, such as a large surface area, surface modification capability, and the ability to control size and shape. These properties contribute to increased drug transport and release efficiency, making them attractive candidates as carriers in drug delivery therapies [10].

Another significant aspect is the ability of nanomaterials to interact with biological structures at the molecular level. Due to their unique surface properties, nanoparticles can interact with proteins and cells, influencing their bioavailability and distribution in the body. Crossing biological barriers, such as the blood-brain barrier or endothelial barrier, becomes possible due to the specific characteristics of nanomaterials [11].

Compared to other advanced nanomaterials, such as quantum dots, nanomaterials exhibit additional benefits, especially in drug delivery. Despite possessing unique optical properties, quantum dots may have limitations related to toxicity and challenges in controlling their size. On the other hand, while characterized by excellent electrical conductivity, carbon dots may be challenging to functionalize for specific biochemical properties. In the case of nanomaterials, owing to their versatility, there is the potential to employ diverse drug delivery strategies tailored to specific therapeutic needs [12].

In a scientific article, Fleige et al. [13] discuss the growing interest in utilizing polymeric nanocarriers to transport active compounds such as small-molecule drugs, peptides, and genes. The application of these nanocarriers aims to enhance the properties of existing drugs, including solubility, bioavailability, and prolonged circulation time, while also allowing customization to individual needs through the selective release of the payload at specific sites of action.

Nanocarriers, also referred to as intelligent drug delivery systems, are designed to respond to specific stimuli such as pH, temperature, redox potential, enzymes, light, and ultrasound, enabling a controlled release of the active substance. In the case of biological stimuli, such as variations in pH within different cellular compartments, nanocarriers respond naturally. For diseases like tumors, tissue differences in pH and temperature can be exploited for targeted drug release. Examples of such systems can also be found in supramolecular drug delivery systems based on polymeric core-shell architectures [14].

Furthermore, external stimuli like light and ultrasound allow for temporal and spatial control of drug release, which is crucial in therapeutic applications, as it is independent of biological events. The author emphasizes the significance of these advanced nanocarrier technologies, which have the potential to revolutionize drug therapy by delivering active substances in a precise manner, minimizing side effects, and enhancing treatment efficacy.

In summary, nanomaterials, as drug carriers, represent an innovative approach to pharmaceutical therapy, harnessing the benefits of nanotechnology to improve drug delivery effectiveness, precision, and safety. A fascinating group of nano-sized carriers are nanoparticles of selected biomaterials, as well as nanocomposites fabricated using biomaterials [15]. In today’s era of medicine, nano-based drug delivery tools are gaining increasing interest due to their potential to address issues associated with traditional therapeutic substance administration methods [16]. The utilization of biomaterials as carriers in conjunction with drugs is the subject of intensive research, representing a direction that could revolutionize the field of pharmaceutical therapy [17].

**Figure 2:**
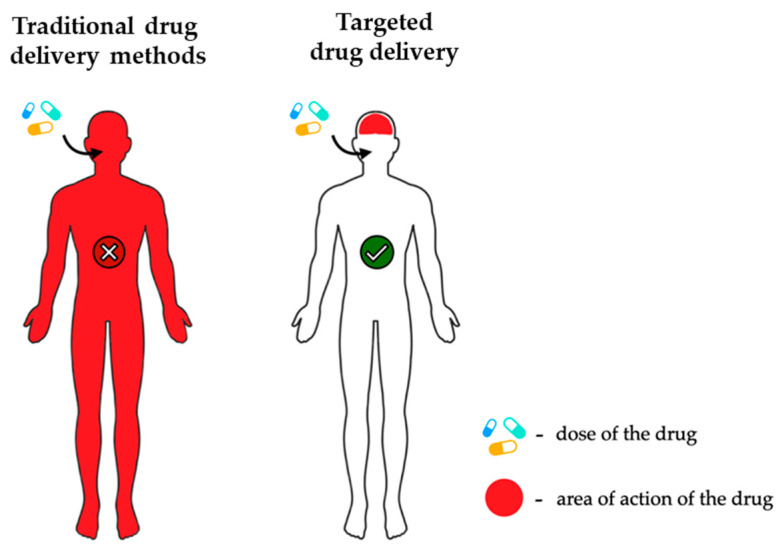
Comparison of traditional and targeted methods in drug delivery.

One key advantage of combining biomaterials with drugs is their ability to precisely deliver therapeutic substances to the body [18]. An example illustrating such drug action is presented in the following figure (Figure 2).

Drugs combined with biomaterials allow for controlled release, increasing therapeutic efficacy and minimizing side effects [19,20]. The application of nanotechnology improves the bioavailability of therapeutic substances and eliminates issues related to their nonspecific distribution in the body [21].

Introducing therapeutic substances into the body without the support of nanotechnological carriers poses specific challenges and drawbacks. Traditional methods of drug delivery often result in low bioavailability and uncontrolled release of substances, leading to toxicity and side effects on normal cells and tissues [22,23]. Additionally, in the case of anticancer therapy, conventional drugs often damage healthy cells, generating undesirable side effects [24]. Specifically, anticancer treatment may be associated with side effects such as tissue damage, a weakened immune system, and drug resistance. Exploring new drug delivery strategies using nanotechnology and biomaterials is a crucial area of research aiming to enhance therapeutic efficacy and minimize adverse side effects [25,26]. However, despite significant progress in nanoscale drug delivery, a scientific niche focuses on improving the precision, effectiveness, and safety of pharmaceutical therapy [27]. This niche encompasses emerging areas of nanomedicine, nanopharmacology, and biomaterial engineering [28].

Introducing biomaterials as nanoscale drug carriers brings a new perspective to this scientific area, focusing on designing biocompatible materials that can be effectively utilized for targeted drug delivery [29]. Combining biomaterials with nanofillers creates platforms that enhance the stability and control of drug release, minimize side effects, and increase their bioavailability [30].

In this article, we will examine current challenges and the quest for new solutions in pharmaceutical therapy, emphasizing minimizing side effects and increasing the precision of drug delivery [31,32]. This literature review plays a crucial role in understanding and emphasizing the significance of biomaterials in nanoscale drug delivery. Our main purpose was to characterize nano-sized biomaterials conjugated with drugs acting as nanocarriers (such as chitosan nanoparticles), drug delivery systems based on nanocomposites fabricated using biomaterials, as well as biomaterials incorporated with nanocomponents as drug carriers. Due to the extensive scope of this research subject, this paper is focused primarily on combinations of the mentioned structures with drugs.

**Figure 3:**
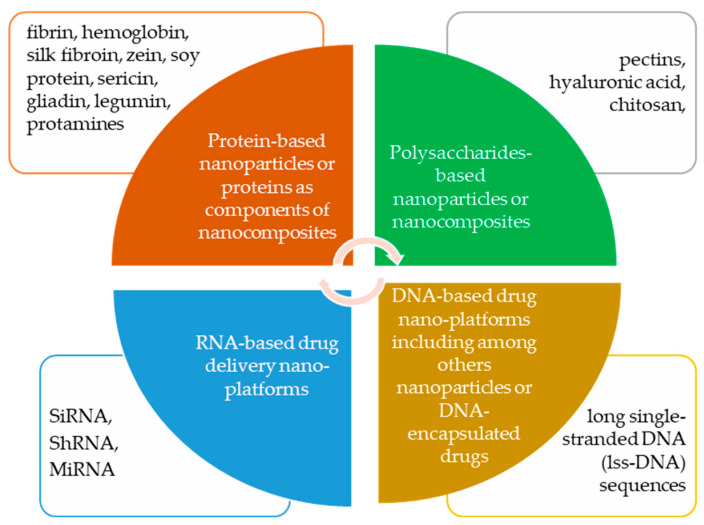
Compilation of biomaterials used to fabricate drug delivery platforms discussed in this review paper.

Our literature review is innovative compared to other publications of this kind because it addresses the combinations of selected biomaterials with drugs in the context of nanotechnology (nano-sized drug delivery platforms). Importantly, we have primarily focused on the application of these combinations in the delivery of anticancer drugs. Figure 3 illustrates the biomaterials described in this review that are utilized in developing drug delivery platforms.

The article belongs to a series of publications on nano-sized drug delivery platforms developed using biomaterials. The first part of the series, which describes—among other things—the role of lipids, proteins such as gelatin or albumin, and polysaccharides such as alginates or cellulose, has already been published [33].

This is important for several reasons. Firstly, the emerging fields of nanomedicine, nanopharmacology, and biomaterial engineering are critical areas of research aimed at improving pharmaceutical therapy. In this context, a review of this topic provides current information on the latest scientific achievements, which can lead to further innovations and progress in the field [34]. 

Secondly, combining nanomaterials with drugs opens new perspectives in designing nanoscale drug carriers, which is essential for improving pharmaceutical therapy’s precision, effectiveness, and safety. This review focuses on analyzing diverse biomaterials and their applications, contributing to identifying the best practices in designing modern drug delivery systems [35]. 

Thirdly, this topic is directly relevant to the search for alternative solutions in pharmaceutical therapy, especially to minimizing the side effects of anticancer treatment and other medical fields. Reviewing the significance of this combination allows for a better understanding of how biomaterials with nanofillers can be effectively used to improve existing treatment methods [36]. 

Ultimately, this literature review significantly contributes to the evolving fields of applied nanotechnology in medicine and inspires further research into innovative drug delivery strategies on a nano scale.

## 2. Proteins in Drug Delivery Nanoscale Systems

Proteins, as essential biological macromolecules, play a significant role in cell structure and function. Chemically, they are polymers of amino acids linked by peptide bonds. Depending on the sequence of amino acids and their arrangement, proteins adopt a specific tertiary structure that determines their function and properties. Proteins have a variety of functions, from enzymatic to structural and transport. They are known for their ability to bind chemicals, catalyze biochemical reactions and interact with other molecules. Moreover, their role in regulating biological processes makes them essential to the life of organisms [37,38].

Proteins have unique properties that make them promising drug carriers, especially in the form of nanoparticles and components of nanocomposite carriers. Their ability to specifically bind active substances enables the precise delivery of drugs to specific sites in the body. The tertiary structure of proteins allows the design of nanoparticles with a diverse surface area, which affects their interactions with target cells [39,40]. In addition, proteins often exhibit biocompatibility, minimizing immune reactions and toxicity. The possibility of chemical modifications allows for controlled drug release, increasing the effectiveness of therapies. These features make proteins a promising tool in drug carrier engineering, enabling the development of innovative therapeutic strategies in nanomedicine [41,42].

The fabrication of protein nanoparticles for use in controlled drug delivery requires fine-tuning a number of their parameters, i.e., size, morphology and selected physicochemical properties, including surface properties. A vital aspect of the controlled design is manipulating protein self-organization processes to achieve the desired morphology. Surface modification of protein nanoparticles affects their physicochemical properties, bioavailability, and stability in the body. Surface functionalization of protein nanoparticles, via—for example—adding selected chemical groups such as thiol, amino, or carboxyl, allows the attachment of a variety of molecules to the surface of proteins, which improves their stability, reduces aggregation, and enables controlled drug release [43,44]. 

A specific type of such a molecule is called a ligand. They are crucial in controlled drug delivery via protein carriers, enabling particular interactions with target cells. These small molecules or chemical groups attached to the surface of protein nanoparticles can significantly affect biodistribution, cell penetration, and selectivity of drug delivery. Ligands are designed to interact with receptors on the surface of target cells specifically. This type of receptor recognition enables protein nanoparticles to target drug delivery to specific cells, which minimizes side effects and increases therapeutic efficacy [45,46,47].

In addition, ligands can affect the bioavailability of protein nanoparticles, mainly by reducing their recognition by the immune system, thereby limiting the immune response. For example, modifying the surface of proteins with poly(ethylene glycol) (PEG) can minimize the immune system response, which increases the circulation time of such carriers and the efficiency of drug delivery [48,49]. Moreover, ligands can promote nanoparticle penetration across biological barriers, such as the blood-brain barrier. Designing ligands that can cross these barriers in a targeted manner may be crucial for delivering drugs to anatomical areas that are difficult to access [50,51]. Examples of widely used ligands are, among others, folic acid and biotin [52], arginine-glycine-aspartate (RGD) peptide [53], transferrin [54], or hyaluronic acid [55].

Maintaining their ability to carry and release drugs is essential when designing protein nanoparticles for controlled drug delivery applications. This allows protein nanoparticles to be tailored for specific drug therapy applications, increasing efficacy and minimizing side effects. The morphology aspect of protein nanoparticles plays an essential role in their design, as the shape and morphological structure affect many fundamental properties, such as surface-to-volume ratio, stability, and the ability to interact with target cells. Fine-tuning the morphology of protein nanoparticles allows for optimal drug delivery use. In addition, one crucial morphological aspect is the control of nanoparticle size. The size of nanoparticles affects their bioavailability, ability to penetrate target tissues, and physicochemical stability. In turn, the shape of nanoparticles affects biological activity. For example, nanoparticles with a spherical shape may have better blood circulation properties than nanoparticles with an irregular shape. The shape may also influence the mechanisms of cellular endocytosis, which is essential for the uptake of nanoparticles by target cells [56,57,58].

In the following subsections, examples of drug carriers, including protein nanoparticle carriers or protein-based nanocomposite carriers, have been presented. The attention has been directed towards two proteins, i.e., fibrin and pectin.

### 2.1. Application of Fibrin in Drug Delivery Nanoscale Systems

Fibrin is a blood protein that plays a crucial role in clotting. Chemically, it is a product of fibrinogen, which converts to fibrin under the influence of the enzyme thrombin. Fibrin is essential for forming a blood clot, and its fibrils form the main structure of the clot. Its ability to create a three-dimensional mesh of bonds allows platelets to stick together, forming a stable clot. Fibrin is a critical element in wound healing and preventing excessive bleeding. In medicine, fibrinogen preparations treat hemorrhages [59,60].

Fibrin, due to its unique properties, shows application potential and is used in medicine, especially in drug delivery systems. Its ability to form a highly stable three-dimensional structure makes it an attractive material for fabricating drug carriers. Fibrin may be modified to control the release of active substances, which is crucial in drug therapy. Its natural biocompatibility reduces potential immune reactions, making it safe for the body. In addition, fibrin can interact with cells, which can be used for targeted drug delivery to specific areas [61,62].

Known for its hemostatic and proangiogenic properties, fibrin is commonly used in various forms, such as disks, films, gels, spheres and nanoparticles. All fibrin forms convert to gel once water is delivered and absorbed. Biodegradability and biocompatibility are critical advantages of fibrin, allowing implantation of the product without the need to remove the carrier after drug release. In medicine, fibrin-based drug delivery systems are widely proposed and used, especially in treating wounds, infections, and cancerous conditions [62].

Many investigations are being conducted on fibrin nanoparticles as drug delivery systems. For example, Vedakumari et al. [63] synthesized fibrin nanoparticles combined with fluorescein isothiocyanate for cellular uptake and further in vivo biodistribution investigations. The performed synthesis led to the preparation of spherical fibrin nanoparticles with sizes within the 25–28 nm range. Moreover, the biocompatibility of formulated nanomaterials and their non-cytotoxicity towards tested cell lines were confirmed. The studies also demonstrated that the cellular uptake of fibrin nanoparticles and their blood circulation time was adequate in terms of their potential applications as drug delivery systems. In vivo, experiments also proved the relative non-toxicity of developed materials, which was concluded based on histopathology analysis, serum biochemistry, and hematology results. Muhamed et el. have investigated fibrin nanoparticles as growth factor delivery systems [64]. In these studies, fibrin nanoparticles have been obtained using a microfluid droplet and subsequently combined with keratinocyte growth factor (KGF). It was demonstrated that developed carriers coupled with KGF enhanced in vitro cell migration, supported cell adhesion, and thus the in vivo wound healing processes. Importantly, it was proved that developed systems showed more advantages than the components (fibrin nanoparticles and KGF) applied separately. In another work, Praveen et al. [65] described studies on fibrin nanostructures—such as fibrin nanoparticles and nanotubes—prepared via the modified water-in-oil emulsification diffusion method. Formulated nanomaterials showed high stability and were investigated mainly regarding the sustained delivery of tacrolimus (an immunosuppressive drug).

Based on the performed studies, a relatively high encapsulation efficiency (66%) was determined, as well as the sustained drug release capability of obtained nanosystems. It was demonstrated that a complete release of tacrolimus took place over one week, wherein the study was carried out in an environment with pH = 7.4. In the case of studies performed in more acidic environments, the drug release also occurred, but the process was slower. Importantly, in vivo studies performed using Sprague Dawley rats confirmed the sustained tacrolimus release ability for both parenteral and oral delivery routes. In turn, Alphonsa et al. [66] verified the potential of fibrin nanoparticles in the delivery of antimicrobial drugs as Ciprofloxacin and Fluconazole. Firstly, fibrin nanoparticles were obtained via the oil-in-water emulsification-diffusion method by adding thrombin to the aqueous suspension of fibrinogen to trigger the crosslinking process. Both drugs were previously introduced into the fibrinogen suspension; as a result, obtained fibrin nanoparticles were simultaneously loaded with active antimicrobial substances. Release investigations showed a significantly higher drug release ability of formulated nanoparticles in an environment with pH = 8.6 than in pH = 7.4. Importantly, in both tested media, a higher release rate of ciprofloxacin was observed. Moreover, both drug-loaded fibrin nanoparticles demonstrated good antifungal and antibacterial properties and non-cytotoxicity towards human dermal fibroblasts.

Many studies have also been focused on fibrin-containing nanocomposites verified for sustained drug delivery. For example, Sundaram et al. [67] fabricated chitin/fibrin nanocomposite gels with gelatin nanoparticles loaded with tigecycline (an antibiotic). Formed nanocomposite gels showed cytocompatibility towards tested cells (human umbilical vein endothelial cell lines), sustained drug release ability (21 days), and in vitro antibacterial activity. Additionally, rapid blood clotting potential of formulated nanocomposite gels (e.g., the hemostasis under pressured femoral artery bleeding conditions was achieved within 154 s) was also reported.

Other examples of drug-delivery fibrin-based nanoscale systems or fibrin-containing nanocomposites have been presented below in Table 1.

Fibrin, due to its ability to form a three-dimensional structure and biocompatibility, plays a crucial role in drug delivery. Its properties make it possible to construct drug carriers, especially fibrin nanoparticles and fibrin-containing nanocomposites, opening up prospects for innovative therapeutic strategies in the medical field.

### 2.2. Application of Hemoglobin in Nanoscale Systems Developing for Active Substance Delivery

Hemoglobin is a metalloprotein complex found in vertebrate blood erythrocytes and is responsible for transporting oxygen and carbon dioxide [72]. Chemically, hemoglobin consists of four globin subunits bound to iron-containing heme groups. Its quaternary structure allows a high affinity for oxygen [73]. Hemoglobin manifests the ability to cooperatively combine and release oxygen depending on the oxygen concentration in the environment [74].

Hemoglobin finds applications in medicine, particularly as a potential drug carrier. Its ability to bind and transport oxygen suggests the potential for use as an oxygen therapy carrier to treat tissue hypoxia [75]. Additionally, the flexibility of hemoglobin’s structure allows for chemical modifications, enabling controlled drug release [76]. Hemoglobin, a natural gas transporter, can be adapted for targeted delivery of active substances to specific body areas. Its biocompatibility and ability to mimic physiological processes make it an attractive candidate for developing innovative drug carriers, especially in the context of gas-transport-related therapies [77].

One excellent interest is in developing practical and safe synthetic oxygen carriers. Hemoglobin seems an ideal candidate for this purpose due to its oxygen-transport capability; thus, many investigations are currently being conducted on such hemoglobin-based oxygen nanocarriers [78]. For example, Liu et al. [79] presented hemoglobin nanoparticles obtained via the electrospray technique, which were coated with self-polymerized and antioxidant polydopamine. This aimed at minimizing the hemoglobin conversion into its nonfunctional oxidized form—methemoglobin. Moreover, such a formed structure was subsequently functionalized using PEG. Performed experiments allowed us to conclude that formulated functionalized nanoparticles showed bio- and hemocompatibility. Importantly, conducting surface functionalization using PEG resulted in lower protein adsorption on the nanoparticle’s surface and, thus, a prolonged retention time within the bloodstream. It was also reported that the presence of PEG and polydopamine affected neither the reversible oxygen-binding of hemoglobin nor its releasing properties. Hence, it was finally concluded that promising oxygen carriers that could be used as a synthetic blood substitute have been developed. Similar investigations have been performed also by Wang et al. [80]. In these studies, poly(ethylene glycol)-functionalized hemoglobin was developed as an efficient oxygen carrier.

Particular attention was also paid to applying hemoglobin-based nanocarriers as tools supporting cancer treatment [81]. One potential strategy for optimizing the effectiveness of current therapeutic procedures used to treat cancer is to increase oxygen perfusion in neoplastic tissues. Although such an intervention may promote faster progression of tumor growth, it is speculated that a concomitant increase in the metabolic activity of tumor cells may affect their susceptibility to conventional chemotherapy and radiotherapy [82]. Jiang et al. [83] performed studies on hemoglobin-linked conjugated poly[2-methoxy-5-(2-ethylhexyloxy)-1,4-phenylenevinylene] nanoparticles. These polymer nanoparticles may absorb the luminol chemiluminescence and thus sensitize the oxygen delivered by hemoglobin to form reactive oxygen species, being toxic towards cancer cells. This, combined with simultaneous delivery of cytostatic drugs, may increase the effectiveness of treatment. In turn, Zhao et al. [84] presented studies on poly(ethylene glycol)-functionalized hemoglobin nanoparticles incorporated with paclitaxel (cytostatic drug). Based on in vivo experiments, it was concluded that formulated nanoparticles accumulated within the tumor tissues and demonstrated anticancer activity.

Moreover, it was stated that the anticancer activity of developed formulations was higher than in parallel analyzed commercial formulations. In another work [85], a near-infrared dye (IR780) was encapsulated into hemoglobin nanoparticles, wherein such a formulated system was considered an oral administration drug during in vivo studies. Studies demonstrated high stability of nanoparticles both in acidic and enzymatic conditions. Furthermore, it was also reported that IR780 effectively accumulated within the tumor tissue and caused a photothermal effect. This, in turn, led to tumor ablation after oral administration of developed nanoparticles in mice affected by cancer.

Hemoglobin-based nanocarriers may also play an essential role in inflammatory bowel disease treatment. Vaezi et al. [86] developed hemoglobin nanoparticles conjugated with 5-aminosalicylic acid (a drug showing anti-inflammatory activity). It was reported that formulated systems did not undergo enzymatic and chemical hydrolysis in simulated body fluid for over 6 h. Importantly, prolonged release of anti-inflammatory drugs for over 72 h was observed. The permeability of formulated nanocarriers in intestinal epithelial cells and their mucus adhesion properties make them promising candidates for colonic drug delivery applications.

### 2.3. Application of Other Proteins in Nanoscale Systems Developing for Active Substance Delivery

Many proteins are currently being investigated regarding their potential applications for sustained active substance delivery. A high potential of such proteins as silk fibroin [87] or sericin [88] has been observed. The examples of such studies, including analyzed proteins, active substances, and the structure of nanoscale delivering systems, have been presented below in Table 2.

The properties of proteins make them beneficial materials widely applied for fabricating active substance carriers. Another biopolymer considered to be a promising tool within drug delivery applications is DNA. The next section of this review paper presents examples of DNA-containing nanoscale delivery systems.

## 3. Nucleic-Acid-Based Drug Delivery Systems

**Figure 4:**
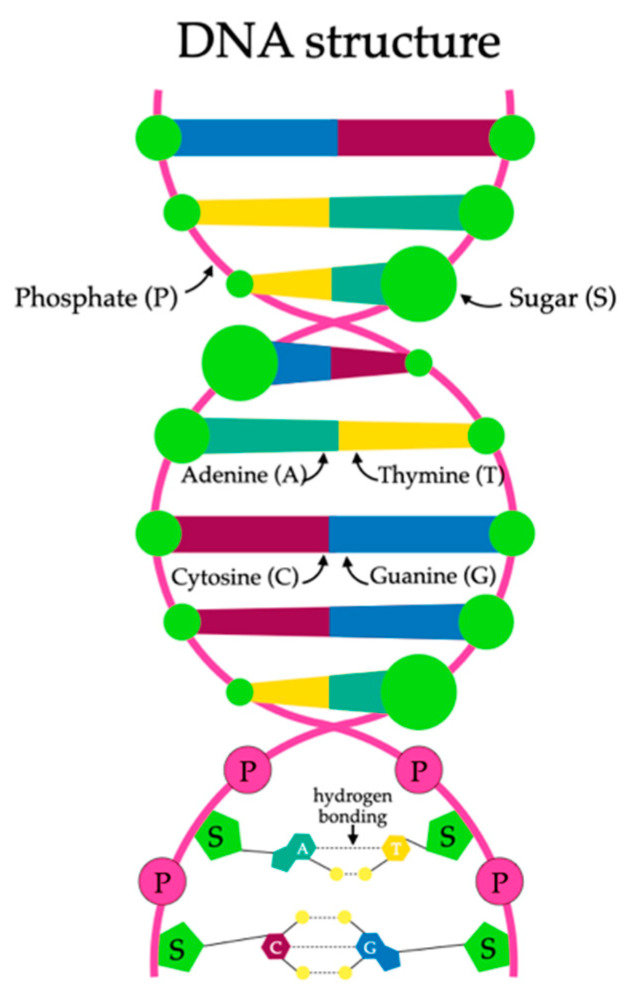
The structure of the deoxyribonucleic acid molecule (DNA).

DNA, or deoxyribonucleic acid, constitutes the fundamental unit of genetic inheritance in all known organisms [125]. It is a complex biopolymer composed of two chains of nucleotides intertwined in a characteristic double helix [126]. Its structure is depicted in the figure below (Figure 4). The function of DNA is not limited solely to the transmission of genetic information, but also holds significance in the context of innovative drug carriers [127].

The role of DNA in drug carriers, especially in nanotechnology, is becoming increasingly significant. Using DNA-based materials to construct drug carriers aims to improve therapeutic effectiveness and minimize side effects [128]. One approach involves integrating DNA with nanomaterials such as nanoparticles or nanotubes to create carriers with controlled drug release [129].

In the case of DNA-based carriers, these nanoparticles can serve as a platform for transporting drugs to specific cells or tissues. The created structures can be tailored to specific requirements, enabling the precise delivery of therapeutic substances to their target locations [130]. Additionally, due to DNA’s ability to interact with various particles, it is also possible to enhance the stability and bioavailability of drug carriers [131].

For example, constructing DNA-based nanoparticles allows for controlled drug release in response to specific environmental conditions, increasing the precision of therapy [132]. Furthermore, introducing specific DNA sequences that interact with particular targets in the body is possible, enabling increased selectivity and therapeutic effectiveness [133].

**Figure 5:**
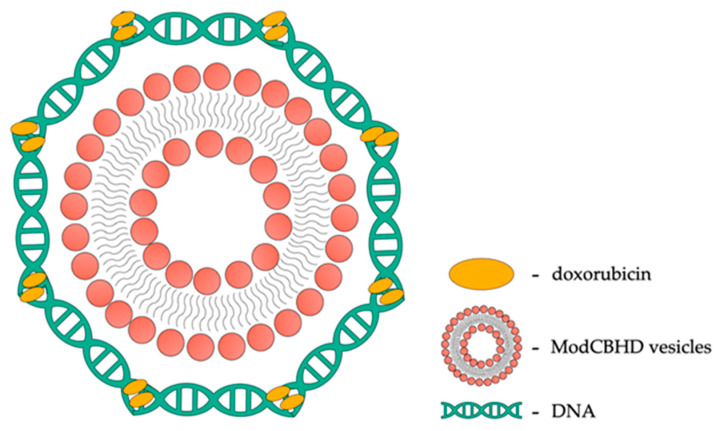
Cationic vesicles decorated with DOX-DNA.

For example, doxorubicin (DOX) is an effective chemotherapeutic drug, but its nonspecific distribution in the body can lead to side effects on normal tissues. Therefore, there is an urgent need to develop drug delivery systems for therapeutic sites with limited side effects. The Alarcon team [134] conducted research to assess the interaction between Modified Cyclodextrin-Based Hollow Vesicles (ModCBHD) vesicles based on cyclodextrins and the DNA–DOX complex as a carrier for anticancer drugs. An example of such a combination is presented in the illustration below (Figure 5).

The results showed that ModCBHD vesicles, with a positive surface charge, enable the wrapping of negatively charged DNA-DOX complexes. Additionally, DOX interacts with DNA through intercalation and ionic interactions. ModCBHD-DNA-DOX complexes exhibit favorable characteristics, such as small size and uniform distribution, which can be utilized in cancer therapy due to the enhanced permeability and retention (EPR) effect. The release of DOX from ModCBHD-DNA-DOX was slower than from DNA–DOX, which is associated with the formation of external complexes after partial intercalation of DOX. The study’s overall conclusions highlight an innovative strategy combining vesicles with cyclodextrins and DNA as alternative carriers for chemotherapeutic drugs, such as Dox, with a delayed release.

Other researchers, such as the Ito team [135], have focused on developing DNA supramolecules based on hydrophobic interactions as effective carriers for cytosine-phosphate-guanosine (CpG) DNA to immune cells. The interaction of unmethylated CpG with mammalian immune cells through Toll-like receptor 9 (TLR9) forms the basis for effective adjuvants in treating immunologic and allergic diseases. However, challenges exist related to low stability in the presence of DNase and limited efficiency in delivering CpG DNA to immune cells. To overcome these challenges, innovative DNA supramolecules were proposed based on long single-stranded DNA sequences (lss-DNA) synthesized using rolling circle amplification (RCA) and cholesterol-modified DNA (chol-DNA).

Microscopic studies revealed that the mixture of lss-DNA with chol-DNA formed micrometer-sized supramolecular structures. This formed DNA gained stability in the presence of DNase compared to lss-DNA, as verified by experiments using fetal bovine serum (FBS). Importantly, it was found that DNA supramolecules induced a three times higher TNF-α release from RAW264.7 cells than lss-DNA alone.

As part of the research into novel approaches to constructing amphiphilic drug complexes, the Yan Zhao team [136] developed unique DNA structures where hydrophobic drug patterns (HDPs) were precisely programmed. The goal was to investigate how these asymmetric HDPs affect drug uptake efficiency by cells and their therapeutic effectiveness, with a particular focus on cytotoxicity against cancer cells.

Experimental results demonstrated that asymmetric hydrophobic drug patterns created on DNA structures significantly increased cytotoxicity against cancer cells. This effect indicates the promising potential of a new approach to designing drug complexes, where precise DNA structure programming can significantly impact therapeutic efficacy. These findings contribute significantly to the evolving nanomedicine and cancer therapy fields, opening new perspectives in designing innovative amphiphilic drug carriers.

As part of research on innovative DNA nanocarriers, the team led by Christine G. Oster [137] developed unique structures based on biodegradable polyesters involving amino-modified poly(vinyl alcohol) (PVAL) frameworks anchored with PLGA groups.

These high-molecular-weight biodegradable polyesters exhibit specific characteristics, such as electrostatic interactions between DNA and cationic branched polyesters, facilitating the loading of DNA nanocarriers. The obtained nanocarriers demonstrated promising morphological parameters, and DNA was released as intact supercoils.

Biological studies confirmed the effectiveness of DNA delivery by these nanocarriers. In in vitro transfection tests on four cell lines, gene delivery using amino-modified polymers was more effective than with unmodified DNA. The type of amine and the distance of the cationic charge from the polymer backbone significantly influenced efficiency.

Experiments with luciferase expression confirmed that DNA nanocarriers exhibited higher transfection efficiency than other methods, such as complexes with PEI 25 kDa. These promising results encourage further research into the application of these DNA nanocarriers in gene therapy, especially in the context of DNA vaccines, which is the subject of ongoing study by Christine G. Oster’s team.

Hydrogels are of interest in biomedical applications, such as tissue engineering or drug delivery, due to their unique properties, such as porosity, high water content, softness, and biocompatibility. This paragraph presents an innovative drug delivery system based on Silica Nanoparticle/Carbon Nanotube–DNA (SiNP/CNT–DNA) nanocomposites, synthesized modularly using carbon nanotubes functionalized with DNA and silica nanoparticles through enzymatic rolling circle amplification. Specific molecular recognition properties were introduced through the design of DNA sequences, resulting in Guanine-Cytosine/Cytosine-Guanine (GC/CG) loop motifs and aptamers enabling the selective binding of intercalating drugs and cell surface receptors.

In conceptual studies, Yong Hu and Christof M. Niemeyer [138] used this system, directing drug-loaded nanocomposites with anthracycline to HeLa cancer cells. Observations suggest that these designed materials act more effectively than the pure therapeutic substance alone, opening perspectives for further research into selectively activating more complex cellular pathways. The results of these studies represent a step towards developing advanced drug carriers, and the designed nanocomposites may find applications in targeted and controlled drug delivery, as well as DNA intercalators.

Let us consider the second of the nucleic acids, RNA, or ribonucleic acid. It is a pivotal element in cell biology, essential in transmitting and regulating genetic information [139]. Composed of nucleotides, including ribose, phosphate groups, and nitrogenous bases, RNA differs from DNA in terms of chemical structure and function. Within cells, messenger RNA (mRNA) conveys genetic information from DNA to ribosomes, where protein synthesis occurs. At the same time, ribosomal RNA (rRNA) forms the structures of ribosomes, and transfer RNA (tRNA) transports amino acids to the sites of protein synthesis. Additionally, various types of RNA, such as small interfering RNA (siRNA) and microRNA (miRNA), play a crucial role in gene expression regulation [140]. Understanding the role and functions of RNA is vital for progress in molecular biology and gene therapy.

In the realm of drug delivery therapies, RNA plays a fundamental role, particularly in the context of gene therapy. The utilization of small RNA fragments, such as siRNA or miRNA, enables precise regulation of gene expression associated with pathologies. This method allows for temporarily silencing or blocking specific genes, becoming a key aspect in treating genetic diseases and cancers [141]. Among gene therapy technologies, antisense oligonucleotides (ASOs) represent another area of RNA application. These short nucleotide sequences, complementary to specific mRNA fragments, can effectively inhibit translational processes or initiate protein synthesis, leading to the blockade of a particular gene expression [142]. An up-and-coming field also uses RNA itself, namely mRNA, as a potential therapeutic agent. In mRNA technology, RNA acts as a carrier of genetic information, delivering instructions to cells regarding producing specific proteins. This approach finds application in treating cancers and genetic diseases [143]. In the context of vaccines, mRNA-based vaccines present an innovative approach. mRNA delivers genetic information that initiates the production of viral proteins or their fragments in cells, inducing an immune response [144]. The efficacy of RNA therapy can be further enhanced by using nanoparticles or carriers, such as liposomes or chitosan nanoparticles. These carriers aim to increase the stability of RNA, protect it from degradation, and enable targeted delivery to specific cells or tissues [145].

In summary, using RNA in drug delivery therapies represents a dynamically evolving field, introducing innovative strategies for treating diverse pathologies. However, despite advancements, further research is required to refine these therapies’ effectiveness, safety, and stability.

The remaining connections and conclusions from the research are presented in the table below (Table 3).

**Figure 6:**
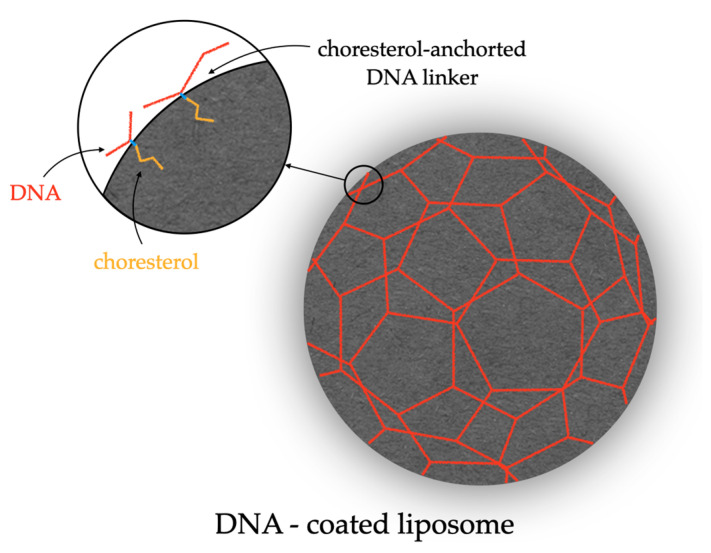
Scheme of DNA-Modified Liposomes.

This chapter provides a comprehensive overview of DNA- and RNA-based carriers tailored for various applications in biomedical nanotechnology. An example of such a connection can be seen in the following figure (Figure 6).

Fundamental properties, such as stability, functionalization capabilities, and the controlled delivery and release of bioactive compounds, are discussed. Various design strategies for DNA- and RNA-based carriers are presented, considering both structural and chemical aspects. The analysis of diverse applications in gene therapy, drug delivery, and diagnostics underscores the versatility and potential of these carriers in medicine. The knowledge gained can be harnessed to refine DNA and RNA carrier design further and expand their scope in future biomedical research and innovations.

## 4. Polysaccharides in Drug Delivery Nanoscale Systems

Polysaccharides constitute a significant group of compounds in biology, chemistry, and medicine, playing crucial roles in living organisms [162]. This article will focus on the characteristics of selected polysaccharides, namely pectin, hyaluronic acid, and chitosan. Each of these polysaccharides represents a unique chemical structure and exhibits specific properties that find applications in various scientific and industrial fields [163].

Analyzing these polysaccharides will enhance our understanding of their structure, functions, and potential applications in scientific and practical domains. In the subsequent sections of the article, we will delve into each of these biopolymers, highlighting their significance in the context of modern scientific advancements and innovative therapeutic approaches.

### 4.1. Application of Pectins in Drug Delivery Nanoscale Systems

Pectins, plant substances primarily present in plant cells, including fruits and vegetables, have the ability to form gels. In the food industry, they serve as thickening and gelling agents, and their presence may impact reducing cholesterol levels in the blood [164,165].

Researchers led by Chengzheng Wang [166] have developed apple pectin-encapsulated Fe_3_O_4_ nanoparticles (Fe_3_O_4_/Pectin NPs) through a one-pot synthesis involving the co-precipitation of Fe(II)/(III) ions in an alkaline solution mediated by pectin under ultrasound conditions. This process led to the formation of magnetic nanoparticles within the pectin network. Physicochemical characterization, including electron microscopy (SEM and TEM), energy-dispersive X-ray spectroscopy (EDX), vibrating sample magnetometer (VSM), and X-ray diffraction (XRD), was conducted on the synthesized Fe_3_O_4_/Pectin NPs. The in vitro cytotoxic and anti-colorectal cancer effects of biologically synthesized Fe_3_O_4_/Pectin NPs were evaluated against various cancer cell lines. The anti-colorectal cancer properties of Fe_3_O_4_/Pectin NPs demonstrated significant removal of cancer cell lines in a time- and concentration-dependent manner, with IC50 values ranging from 187 to 337 µg/mL. The antioxidant activity of Fe_3_O_4_/Pectin NPs was determined using the DPPH method, showing high antioxidant activity according to the IC50 value. These nanoparticles’ anti-human colorectal cancer effect is attributed to their antioxidant effects.

**Figure 7:**
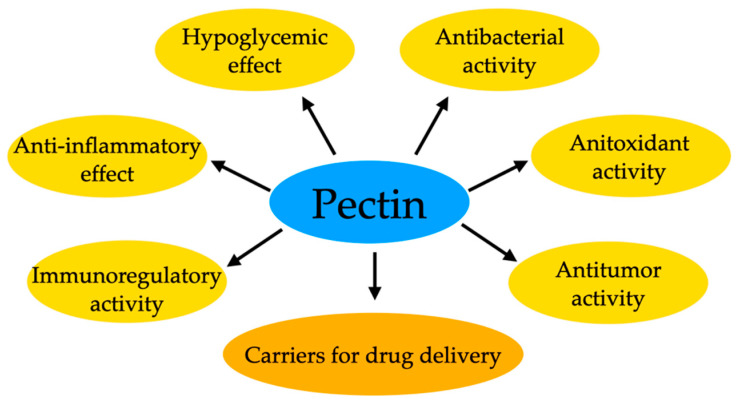
Examples of pectin’s properties as a drug carrier.

Pectin itself has therapeutic properties (Figure 7). Still, researchers are also conducting research that allows obtaining composite materials by combining this biopolymer with a drug. So, for example, hydrogel composites of pectin/bacterial cellulose (BC) with varying BC content were constructed for controlled drug delivery utilizing the conductive polymer polypyrrole. Incorporating polypyrrole allowed drug encapsulation within the pectin/BC composite, enabling controlled release under the influence of an electric field. The release of ibuprofen, serving as a model drug, was investigated, considering the matrix composition, pH stimulation, matrix pore size, and applied electrical potential using a modified Franz diffusion cell. Optimal drug release conditions were achieved at 30% BC by weight, and release increased with applied electrical potential. The highest release efficiency, reaching 78%, was attained for the composite containing polypyrrole under a potential of 7 V. Additionally, the hydrogel composites exhibited significant antibacterial properties against Gram-positive bacteria. These results underscore the potential of pectin/BC hydrogel composites for transdermal drug delivery. The research by the team led by Nattinee Krathumkhet [167] has yielded promising outcomes, opening new avenues for investigations into pectin-based materials for transdermal drug delivery purposes.

The research team led by Arun K. Kodoth [168] conducted a comparative study on the release capacity of Donepezil from a pectin-based gel and its nanocomposite with zinc oxide, aiming for potential use as an implantable drug delivery platform in Alzheimer’s disease treatment. The study analyzed adsorption capabilities concerning pH, temperature, drug concentration, and adsorbent mass. The nanocomposite demonstrated significant adsorption capacity compared to the pure gel. Release kinetics analysis revealed that the nanocomposite followed pseudo-first-order kinetics, while the pure gel exhibited pseudo-second-order kinetics. Polymer samples with adsorbed Donepezil were evaluated for mechanical properties, swelling index, and folding endurance, and characterized using various techniques. In vitro drug release studies showed that the nanocomposite had a desorption capacity of approximately 88%, a notable increase compared to the pure gel (46%). The developed systems also exhibited low hemolysis, suggesting the nanocomposite’s potential for applications in implantable drug delivery systems. These findings represent another example of exploring the combination of pectin with drugs for innovative solutions in drug delivery.

Research on pectin combinations, such as pectin/Fe_3_O_4_, pectin/polypyrrole, or pectin/ZnO, presents a broad spectrum of possibilities for applying this substance in drug delivery. However, considering their diversity, each combination could open up new research areas on pectin as a drug carrier. It would be worthwhile to consider separate studies for each combination to understand their potential and applications fully. Our research aimed to outline this wealth of possibilities, encouraging further investigations into pectin for innovative drug delivery solutions. In the following chapter, we will explore hyaluronic acid as our next subject of consideration.

### 4.2. Application of Hyaluronic Acid in Drug Delivery Nanoscale Systems

Hyaluronic acid, naturally occurring in animals, is critical in maintaining skin hydration, joint elasticity and eye health. Its properties have found applications in cosmetology, aesthetic medicine and the treatment of joint ailments [169,170].

Studies on hyaluronic acid-based nanocarriers applied in breast cancer treatment have been presented by Yu et al. [171]. In this work, hyaluronic acid nanoparticles have been incorporated with both cisplatin and doxorubicin. Performed research demonstrated the sustained drug release ability of formulated nanocarriers, which was the most effective in an acidic environment. Moreover, high cytotoxicity towards cancer cell lines manifested by significant cell growth inhibition was observed. Studies showed that developed nanocarriers demonstrated a stronger inhibitory activity than free cytostatic drugs, thus confirming the application potential of formulated carriers in this field.

In another work [172], innovative reduction-responsive chitosan/hyaluronic acid/lipoic acid-based nanoparticles have been developed to treat breast cancer effectively. Research showed that developed systems were characterized by drug release capability and significant cytotoxicity against breast cancer cell lines. It was also reported that hyaluronic acid played a significant role in biological responses, thus proving the potential of this polysaccharide in drug delivery.

### 4.3. Application of Chitosan in Drug Delivery Nanoscale Systems

Chitosan, a chitin derivative found in crustaceans’ exoskeletons, is an incredibly significant biopolymeric material. Its biodegradability and biocompatibility make it widely applicable in medicine, pharmaceuticals, and the food industry. Serving as a drug carrier, chitosan shows promising prospects in the field of delivering active substances [173,174]. The table below (Table 4) compiles various connections for chitosan-based nanocomposites and drug carriers with selected anticancer drugs and other drugs. The table also highlights the key achievements of scientists involved in developing the described connections.

In summary, the table presents various combinations of drug carriers with chitosan and drug-chitosan nanocomposites that have been tested in different therapeutic applications. The use of diverse nanoparticles such as GTA, CS-NPs, T7-CMCS-BAPE and Ch-IONPs allows for precise drug delivery, finding applications in anticancer therapies and even improving drug accessibility through transdermal routes. Chitosan, serving as a carrier in these nanocomposites, proves to be a promising material in drug delivery, offering efficacy and selectivity in therapeutic applications. These innovative approaches open new perspectives in cancer therapy, drug delivery, and the treatment of neurodegenerative diseases.

## 5. Limitations

Despite the tremendous potential in drug delivery, nanomaterials have challenges and limitations. This chapter analyses key aspects that demand attention when designing nanoparticles as drug carriers. These limitations include potential toxicity, aggregation tendencies, shelf life, and other factors influencing their effectiveness and safety [183].

Depending on their chemical and physical characteristics, Nanoparticles may exhibit toxicity towards cells and tissues. The impact of nanomaterials on human health and the environment requires specific understanding. We delve into current research on the toxicity of nanomaterials and strategies to minimize risk through chemical modifications and surface engineering. For example, studies suggest that modifying the surface properties of nanoparticles with biocompatible coatings can enhance their safety profile [184].

Aggregation of nanoparticles can affect their stability and drug transport capabilities. In designing such materials, we discuss mechanisms leading to nanoparticle aggregation and design strategies aimed at minimizing this phenomenon. Techniques for monitoring and assessing aggregation under laboratory and biological conditions are also analyzed. An example includes stabilizing agents during nanoparticle synthesis to prevent aggregation [185].

The stability of nanoparticles over time is crucial for their efficacy in drug delivery. Factors influencing the shelf life of nanomaterials, such as storage conditions, the influence of the biological environment, and design strategies ensuring prolonged functionality, are examined. It is worth noting that adding stabilizing agents or employing controlled drug release can increase the durability of nanoparticles [186].

Understanding interactions between nanomaterials and cells is crucial for assessing their safety and efficacy. Nanoparticles interact with cell membranes, penetrate cells, and influence endocytosis mechanisms and intracellular drug distribution. An example is targeted nanoparticles designed to enhance cellular uptake and minimize off-target effects [187].

Regarding immunological reactions induced by nanomaterials and their potential biodegradation, achieving a balance between stability and breakdown is crucial for ensuring long-term safety. Studies on the immune system’s response to nanoparticles and the development of biodegradable nanomaterials for environmentally friendly applications serve as examples [188].

For example, Barua and Mitragotri [189] described several significant limitations associated with using nanoparticles to treat various diseases. One major challenge is the ability of nanoparticles to reach the therapeutic site at appropriate doses while minimizing accumulation in other areas of the body. Biodistribution of nanoparticles is subject to various biological barriers, which include immune clearance in organs such as the liver and spleen, as well as permeation through the endothelium into target tissues, penetration through the tissue interstitium, endocytosis in target cells, diffusion through the cytoplasm and, ultimately, entry into the cell nucleus.

In addition, when nanoparticles are delivered through alternative routes, such as the skin and mucous membranes of the nose, lungs, intestines, and vagina, they encounter diffusion resistance in these tissues, which is an essential barrier to the delivery of active substances.

Another significant limitation is that not all nanoparticles can effectively overcome these biological barriers, which may affect their effectiveness as drug carriers. In addition, some NPs delivery strategies may lead to immune system activation, which can trigger adverse immune reactions.

This chapter sheds light on the limitations of nanomaterials that may impact their application in drug delivery. Introducing innovative solutions and design strategies can contribute to overcoming these challenges, paving the way for more effective and safer nanomaterial-based therapies. The literature review conducted, and examples cited from current research, illustrate ongoing efforts to address these issues.

## 6. Conclusions and Perspectives

In conclusion, this comprehensive literature review delves into the pivotal roles of nucleic acids (e.g., DNA), proteins (including fibrin and hemoglobin), and polysaccharides (such as pectin, hyaluronic acid, and chitosan) within contemporary nanoscale drug delivery systems. Examining nanocomposites based on these biomaterials highlights their broad potential applications in drug delivery, particularly in anticancer therapy, showcasing promising outcomes in effectively transporting therapeutic agents to target cells.

As representative examples, pectins, hyaluronan, and chitosan exhibit diverse capabilities in designing innovative carriers, demonstrating effectiveness in eliminating cancer cells and facilitating controlled drug release. Hyaluronic acid, identified as a valuable drug carrier, particularly in anticancer therapy, has shown promise in targeted drug delivery to specific cancer cells, opening avenues for potential clinical applications.

Chitosan, derived from chitin, is a noteworthy biopolymer contributing to nano-level drug delivery. Its versatility, especially when integrated with nanocarriers, showcases potential in targeted drug delivery, proving effective against cancer and bacterial infections.

Additionally, exploring nanocarriers based on hemoglobin introduces novel perspectives, particularly in oxygen therapy and cancer treatment, where enhanced oxygen perfusion in tumor tissues may play a significant role.

These findings underscore the immense potential of nucleic acids, proteins, and polysaccharides as integral components in advancing drug delivery systems. The versatility and effectiveness demonstrated by these biomaterials open avenues for developing innovative therapeutic strategies with broad implications for medical and cancer therapy research.

### Research Perspectives

Further exploration of biomaterial combinations: investigating combinations of pectins, hyaluronic acid, and chitosan with other substances may lead to developing new, effective drug carriers with enhanced selectivity and therapeutic efficacy.

Optimization of nanocarriers in anticancer therapy: continuing research on nanocomposites, especially those based on hemoglobin, aims to optimize their ability to deliver oxygen and drugs to cancerous areas.

Studies on DNA nanocarriers: research on DNA carriers opens new perspectives in gene therapy. Continuing these studies could lead to the construction of more precise and efficient drug delivery systems.

Exploration of novel biomaterials: the search for new biomaterials capable of acting as drug carriers, enhancing biocompatibility, and controlling the release of active substances is essential for future advancements.

These conclusions suggest that further research in nanomedicine and anticancer therapy may bring innovative solutions, improving the effectiveness and selectivity of drug delivery—a crucial aspect for the future of medicine and cancer therapy.

## Figures and Tables

**Table 1:** Drug delivery fibrin-based nanoscale systems or fibrin-containing nanocomposites.

Active Substance	Active Substance Properties	Nanocomposite Structure	Ref.
gallic acid	polyphenolic compound of plant origin showing neuroprotective, anti-inflammatory, anticancer, antioxidant, anti-diabetic, and wound healing properties	fibrin/collagen-based scaffolds incorporated with drug-loaded chitosan nanoparticles obtained via ionotropic gelation	[68]
methotrexate	a drug used among others in inflammatory arthritis treatment may slow down cancer cell proliferation	chitosan/fibrin-based nanocomposites	[69]
tissue-type plasminogen activator (tPA)	thrombotic complications treatment	fibrin/poly(N-isopropylacrylamide)-based nanogels	[70]
tissue-type plasminogen activator (tPA)	thrombotic complications treatment	fibrin-modulating-core-shell poly(N-isopropylacrylamide) nanogels	[71]

**Table 2:** Protein-containing active substance delivery nanosystems.

Protein	Active Substance	Delivery System Structure	Conclusions	Application	Ref.
silk fibroin	curcumin	silk fibroin shelled magnetic nanoparticles (core-shell structure) loaded with a drug (silk fibroin extracted from *Bombyx mori cocoons*)	average particle size: 220 nm or 650 nm (depending on the reagents applied) cytotoxicity of nanocarriers towards human Caucasian breast adenocarcinoma cells and higher cellular uptake the possibility of cancer targeting via the external magnetic field due to the magnetic core	breast cancer treatment	[89]
	naphthalene diimide derivative	silk fibroin nanoparticles functionalized with cyclic pentapeptides incorporating the Arg-Gly-Asp sequence (cRGDs) and loaded with a drug (silk fibroin extracted from *Bombyx mori cocoons*)	average particle size: ~100 nm selectivity in cell uptake and, thus, accumulation within the tumor tissue as a result of functionalization with cRGDs cytotoxicity towards cancer cells	cancer treatment	[90]
	doxorubicin	folate-conjugated silk fibroin nanoparticles loaded with a drug (silk fibroin extracted from *Bombyx mori cocoons*)	average particle size: >200 nm sustained drug release capability of nanocarriers, cancer cell targeting, and cytotoxicity towards them	cancer treatment	[91]
	doxorubicin	poly(ethylene glycol)-functionalized silk fibroin nanoparticles combined with a drug (silk fibroin extracted from *Bombyx mori cocoons*)	average particle size: before PEGylation 104 ± 1.7 nm; after PEGylation: 116.40 ± 3.23 nm high encapsulation efficiency and sustained drug release capability (over 14 days) PEGylated silk nanoparticles significant cytotoxicity towards cancer cells	breast cancer treatment	[92]
	naringenin	drug-loaded silk fibroin nanoparticles (silk fibroin extracted from *Bombyx mori cocoons*)	average particle size: ~150–180 nm high antioxidant activity and cytotoxicity of developed nanocarriers towards cancer cell lines, as well as drug release ability	cancer treatment	[93]
	doxorubicin	core-shell nanoparticles incorporated with a drug, core—drug-loaded silk fibroin nanoparticles; shell—zeolitic imidazolate framework-8 (ZIF-8) (silk fibroin extracted from *Bombyx mori cocoons*)	average particle size: ~180–250 nm high stability of nanocarriers under neutral conditions and their uptake by breast cancer cells, controlled drug release ability due to dissolution of ZIF-8 shells in the acidic intracellular environment, inducing cell apoptosis and thus inhibiting tumor growth by nanocarriers	cancer treatment	[94]
	doxorubicin	PVA/silk fibroin core-shell nanoparticles with drug (silk fibroin extracted from *Bombyx mori cocoons*)	average particle size: 984–1270 nm high encapsulation efficiency and controlled drug release ability of nanocarriers, cytotoxicity of developed nanosystems towards human breast cancer cells whose apoptotic activity increased with time	cancer treatment	[95]
	cisplatin	drug-loaded silk fibroin nanoparticles (silk fibroin extracted from *Bombyx mori cocoons*)	average particle size: 59–75 nm accumulation of developed nanocarriers within the tumor cells, thus triggering their apoptosis sustained drug release ability of nanocarriers	cancer treatment	[96]
	methotrexate	silk fibroin/albumin-based nanoparticles incorporated with a drug (silk fibroin extracted from *Bombyx mori cocoons*)	average particle size: 140–300 nm high encapsulation efficiency and drug release ability (approx. 85% release of active substance after 12 days) non-cytotoxicity of nanocarriers towards fibroblasts and simultaneous cytotoxicity towards human breast cancer adenocarcinoma cells	cancer treatment	[97]
	5-fluorouracil, curcumin	self-assembled silk fibroin nanoparticles incorporated simultaneously with both active substances (silk fibroin extracted from *Bombyx mori cocoons*)	average particle size: 217.0 ± 0.4 nm drug release ability of nanocarriers as well as high loading efficiency noticeable reduction of tumor treated with formulated nanocarriers	breast cancer treatment	[98]
	ascorbic acid	silk fibroin/carboxymethyl chitosan-based composite scaffolds incorporated with drug-loaded chitosan nanoparticles (silk fibroin extracted from *Bombyx mori cocoons*)	average particle size without drug: 133 nm; 199 nm with drug sustained ascorbic acid release capability of formulated scaffolds	bone regeneration	[99]
	doxorubicin	silk fibroin nanoparticles coated with cationic polymers (including glycol chitosan, polyethylenimine, N,N,N-trimethyl chitosan and PEGylated polyethylenimine) incorporated with a drug [core-shell structures] (silk fibroin extracted from *Bombyx mori cocoons*)	average particle size: 168 ± 7 nm accumulation of formulated core-shell nanostructures within the cancer cells cytotoxicity of developed nanocarriers towards human cervical carcinoma cancer cells	cancer treatment	[100]
soy protein	curcumin	drug-loaded soy protein nanoparticles (soy protein isolate was chemically treated and used for further experiments wherein nanoparticles were obtained by the desolvation method)	average particle size: without curcumin: 278 nm; with curcumin: 295 nm slow-release drug ability of nanocarriers cytotoxicity of developed nanoparticles towards osteogenic sarcoma cells	cancer treatment	[101]
	doxorubicin	folic acid (FA)-conjugated soybean protein nanoparticles loaded with a drug (soy protein isolate was chemically treated and used for further experiments, wherein nanoparticles were obtained by the desolvation method)	average particle size: ~206 nm (without FA); 232 nm (conjugated with FA) efficient accumulation of nanoparticles within the tumor area and thus had efficient growth inhibitory capability against multicellular tumor spheroids	cancer treatment	[102]
	vancomycin	drug-loaded soy protein nanoparticles (soy protein was obtained by precipitation via the ammonium sulfate gradient while its nanoparticles were achieved by the desolvation method)	average particle size: without drug: 385.3 nm; with drug: 412.84 nm sustained drug release ability and antibacterial activity of developed nanocarriers	bacterial infections	[103]
	epicatechin, quercetin	drug-loaded inulin–soy protein nanoparticles (soy protein isolate was used for experiments)	average particle size: without drug: 171.0 ± 2.7 nm; with drug: 190.7 ± 2.8 nm sustained drug release ability and cytotoxic effect of nanoparticles towards human colorectal cancer cells	cancer treatment	[104]
	doxorubicin	phenylboronic acid-modified soy protein nanoparticles incorporated with a drug (soy protein was purified and used for the preparation of nanoparticles via the polymer-monomer pair reaction system)	average particle size: 30 nm, 50 nm and 150 nm (depending on the synthesis conditions) the highest cellular uptake and highest cytotoxicity in vitro determined for 30 nm-sized drug-loaded nanoparticles enhanced drug accumulation and the highest in vivo anticancer efficiency determined for 30 nm-sized drug-loaded nanoparticles	cancer treatment	[105]
zein	mometasone furoate	drug-loaded zein nanoparticles	average particle size: without drug: 118 nm; with drug: 100–140 nm the most effective drug release ability in an environment with pH = 6.8	intestinal inflammatory diseases treatment	[106]
	curcumin	dodecamer peptide-functionalized polydopamine-coated drug-loaded zein nanoparticles	average particle size: 50–150 nm (depending on the reaction conditions) high accumulation within the tumor tissue, cytotoxic effect by limiting proliferation and inducing glioma cell apoptosis; ability to circulate, and thus shows the possibility of targeting cancer cells	cancer treatment	[107]
	enrofloxacin, ciprofloxacin, metronidazole, nitrofurantoin, and norfloxacin (antibiotics)	zein nanoparticles	average particle size: 70–95 nm (without drug) drug release capability of nanocarriers in environments simulating gastrointestinal conditions, antibacterial activity	acute promyelocytic leukemia and skin disease treatment, cancer-preventing	[108]
	folic acid	folic acid-linked zein nanoparticles and zein nanoparticles containing physically entrapped folic acid	average size: folic acid covalently linked zein nanoparticles: 67.2 ± 6.6 nm; zein nanoparticles with entrapped folic acid: 96.8 ± 5.9 nm sustained drug release ability of both developed nanosystems (phosphate buffer saline, 7 days, 37 °C)	cancer treatment	[109]
sericin	-	magnesium oxide nanoparticles conjugated with sericin (sericin Bombyx mori (silkworm), S5201)	average nanoparticle size: 65–88 nm anti-ageing, antibacterial, and anticancer activity of developed nanoparticles	anti-aging therapy	[110]
sericin	resveratrol	drug-loaded sericin nanoparticles (sericin extracted from Thai Bombyx mori silk cocoons)	average nanoparticle size: 200–400 nm potent inhibition of cancer cells (colorectal adenocarcinoma) by developed nanoparticles with simultaneous non-cytotoxicity towards skin fibroblasts; sustained resveratrol release for over 72 h	cancer treatment	[111]
sericin	resveratrol, melatonin	sericin-based nanoparticles (sericin from Bombyx mori (silkworm) powder (S5201))	average nanoparticle size: ~128 nm pH-dependent drug release ability of nanoparticles wherein the maximum release in pH = 6 cytotoxicity towards cancer cells	cancer treatment	[112]
sericin	-	sericin-based nanoparticles crosslinked using crocetin (I type) and glutaraldehyde (sericin from Bombyx mori silk cocoons)	average nanoparticle size: 248 nm (nanoparticle crosslinked by crocetin) and 225 nm (nanoparticle crosslinked by glutaraldehyde) antioxidant ability and non-toxicity towards human fibroblasts	neurological disease treatment (via a nose-to-brain delivery system)	[113]
sericin	doxorubicin	surface charge-reversal sericin-based nanoparticles (sericin from Bombyx mori silk cocoons)	average nanoparticle size: 213.0 ± 5.8 nm higher cellular uptake of developed nanoparticles in slightly acidic pH = 6.0 triggered by tumor environment than in pH = 7.4) pH-dependent drug release ability	cancer treatment	[114]
gliadin	usnic acid	gliadin-based nanoparticles conjugated with hyaluronic acid (wheat gliadin was applied)	high cellular uptake and cytotoxic effect towards breast cancer cells effective targeted drug delivery by developed nanosystems	cancer treatment	[115]
gliadin	ascorbic acid	gliadin-based nanoparticles (wheat gliadin was applied)	average particle size: <200 nm high drug loading efficiency of developed nanoparticles prolonged release drug ability both under simulated gastrointestinal conditions and in PBS enhancement of the antioxidant activity of ascorbic acid by gliadin nanoparticles and its protection against UV exposition.	oral nutraceutical delivery	[116]
gliadin	doxorubicin	gliadin nanoparticles (wheat gliadin was applied)	average particle size: ~150 nm high drug loading efficiency sustained drug release ability in media simulating tumor and physiological conditions	cancer treatment	[117]
gliadin	resveratrol	gliadin-based nanoparticles (wheat gliadin was applied)	average particle size: without drug: 121.56 nm; with drug: 236.20 nm high drug encapsulation efficiency of developed carriers sustained drug release ability	bioactive compound delivery in functional beverages and foods	[118]
gliadin	cyclophosphamide	gliadin-based nanoparticles (wheat gliadin was applied)	average particle size: 218.66 ± 5.1 nm gradual drug release from nanoparticles for 48 h cytotoxicity of developed carriers towards breast cancer cells	breast cancer treatment	[119]
legumin	methylene blue	legumin nanoparticles chemically crosslinked with glutaraldehyde (legumin was extracted from pea seed flour)	average particle size: ~250 nm stability of developed nanoparticles in phosphate-buffered saline (PBS) solution loading efficiency of carriers: 6.2% drug release ability	hydrophilic drug delivery	[120]
protamine	rifabutin	protamine nanocapsules	average particle size: ~200 nm nanocapsule stability under storage and in biological media cellular uptake and compatibility with red blood cells drug release ability of nanocapsules	respiratory diseases treatment	[121]
protamine	doxorubicin	chitosan-protamine nanoparticles	average particle size: 117 nm pH-dependent drug release ability, release rates: 60.10%—pH 4.0; 44.15%—pH 6.8 and 25.10%—pH 7.4 cytotoxicity towards breast cancer cells	cancer treatment	[122]
protamine	doxorubicin	nano-complex of protamine and PEG	average particle size: 212 nm sustained drug release at pH 4.8 (intracellular pH of breast cancer cells) cytotoxicity towards breast cancer cells	cancer treatment	[123]
protamine	tacrine	poly-(d,l)-lactide-co-glycolide (PLGA)-based nanoparticles coated with protamine	average particle size: 70.55–237.67 nm entrapment efficiency: 4.35–33.78% sustained drug release for 120 h promising brain-targeting efficiency after intranasal administration	Alzheimer’s disease treatment	[124]

**Table 3:** Examples of combining DNA and RNA with drugs as drug carriers.

Combining	Application	Ref.	Conclusions
DNA-Chitosan Nanoparticles	Combining chitosan with DNA forms a drug carrier, particularly popular for delivering therapeutic genes.	[146]	Chitosan-DNA nanoparticles achieved optimal size and stability with the appropriate ratio of amino and phosphate groups. The nanoparticles demonstrated the ability to protect encapsulated DNA from nuclease degradation. Transfection efficiency was cell-type dependent, with high efficacy observed in HEK293 cells. Chitosan-DNA nanoparticles have the potential to serve as effective gene carriers, and their ability to protect plasmid DNA makes them a promising tool in gene therapy.
DNA-Doxorubicin Complexes	Doxorubicin, an anticancer drug, can form complexes with DNA, creating a drug carrier with enhanced anticancer efficacy.	[147]	The research focused on the interaction between the DNA molecule and the anticancer drug doxorubicin. It was observed that the physicochemistry of the interaction can be modified by changing the ionic strength of the buffer. Under low ionic strength conditions, DOX interacts with DNA through intercalation or the formation of dimeric compounds. At high ionic strengths, an increased spontaneous association of DOX-DOX is observed, leading to the formation of aggregate compounds. The length of persistence of DNA-DOX complexes showed a dependence on force, with different behaviors observed depending on the applied measurement technique.
		[148]	The higher degree of DOX release observed for dipalmitoylphosphatidylcholine (DPPC) was attributed to a stronger lipoplex formation with DNA. DPPC liposomes in the sol-gel phase can absorb more Ca^2+^ ions, enabling electrostatic solid interaction with DNA. The electrostatic interaction was confirmed through time-resolved anisotropy spectroscopy and circular dichroism. Possible hydrophobic interaction between liposomes, and DNA was considered as a contributing factor to the observed deintercalation. Effective uptake of DOX molecules by liposomes from the drug-DNA complex was confirmed using confocal laser scanning microscopy (CLSM).
		[149]	DOX was intercalated with plasmid DNA to form a plasmid DNA/ DOX complex. The plasmid DNA/ DOX complex demonstrated the highest efficacy in inhibiting the proliferation of colon26/Luc cancer cells compared to DOX alone, DNA alone, and other complexes. Plasmid DNA containing a CpG motif (CpG plasmid DNA) was more effective than plasmid DNA without the CpG motif. The delivery of DOX to the tumor tissue in the liver was more significant when using the CpG plasmid DNA/DOX complex than free DOX. The CpG plasmid DNA/ DOX complex more effectively inhibited the proliferation of colon26/Luc cancer cells in the liver compared to other controls.
DNA-Modified Liposomes	Combining liposomes with modulated DNA can create innovative drug carriers, delivering active substances to specific locations.	[150]	Glycol chitosan-coated liposomes show potential as a nasal vaccine delivery vehicle. These liposomes are capable of eliciting significant mucosal and cellular immune responses following intranasal administration to mice, surpassing the response triggered by unmodified DNA. Their ability to protect encapsulated DNA from nucleases and their mucoadhesiveness suggests that they may serve as effective carriers for intranasal vaccine delivery. These findings indicate the potential application of glycol chitosan-coated liposomes in developing nose-delivered vaccines against viruses.
		[151]	Melting transitions (Tm) of these liposomes, depending on lipid charge, size, fluidity, and attached DNA, have been systematically investigated and compared with the properties of gold nanoparticles (AuNPs). There are certain similarities but also significant differences. The Tm of liposome assemblies is less influenced by interparticle separation or liposome size than AuNPs. These fundamental understandings may lead to improved design of new liposome-based materials for various analytical and biomedical applications.
		[152]	The association structures formed by cationic liposomes and DNA have been effectively utilized as gene carriers in transfection assays. The mean size of vesicles increased after the incorporation of DNA into liposomes (673 ± 27 nm). The liposomal formulation significantly enhanced transfection compared to naked DNA as a negative control. Compared with the commercial product Lipofectamine^®^ 2000, the liposomal formula in the study demonstrated better functionality, reflected in higher cellular activity (cellular protein) in the prepared lipoplex than in the case of Lipofectamine^®^ 2000.
		[153]	Small unilamellar vesicles (SUVs) serve as essential model membranes, organelle mimics, and carriers for drugs and vaccines. The lack of robust techniques for functionalizing or organizing performed SUVs limits their applications. Using DNA structures to coat, cluster, and arrange sub-100-nm liposomes allows the generation of distance-controlled networks of vesicles, strings, and dimers, among other configurations. DNA coating also enables the attachment of proteins to liposomes and temporal control of membrane fusion driven by SNARE protein complexes.
DNA-Encapsulated Nanoparticles	Nanoparticles, like lipids or quantum dots, can be surrounded or modified by DNA, providing specific drug carriers.	[154]	DNA vaccines have proven to be an effective antiviral strategy, but biodegradation and adverse environmental conditions challenge their application. A chitosan/sodium alginate (CS/SA) nanoparticle delivery system was employed to develop oral DNA nano vaccines (CS/SA/VP4C NPs and CS/SA/VP56C NPs) to enhance their effectiveness. The DNA nano vaccines had a diameter of approximately 100 nm and a spherical structure, as confirmed by electron microscopy. Administration of the CS/SA/VP4C NPs and CS/SA/VP56C NPs to grass carp significantly increased protective efficacy, with a 42% higher survival rate compared to the control group. Vaccine administration led to a rapid increase in serum immune markers and a significant upregulation of mRNA expression of critical genes associated with immunity. The CS/SA/VP4C + VP56C NPs group exhibited the highest protective efficacy and the ability to stimulate an immune response. The results suggest that oral administration of nano vaccines can effectively prevent grass carp reovirus infections, representing a promising strategy for combating viral diseases in fish.
		[155]	Silica nanoparticles with encapsulated DNA (SPED) represent a promising substitute for simulating pathogen transmission, eliminating the infection risk associated with live organisms. In the studies, SPED demonstrated high adhesion to exposed skin after hand-to-surface contact, spreading SPED1–3 on various materials. This pattern reflected touching sequences consistent with areas contaminated by fingers compared to palms.
		[156]	Nanoparticles enable the storage of various types of therapeutic and diagnostic agents. Controlling the storage of molecules in nanoparticles is challenging due to nonspecific intermolecular interactions used for encapsulation. Specific DNA interactions were employed in the study to store molecules in nanoparticles. Nanoparticles containing DNA anchors were created to capture small molecules conjugated with DNA. The amount and ratios of different nanoparticle molecules can be controlled by altering the sequences and stoichiometry of DNA anchors. Modifying the ratio of encapsulated drugs allows the adjustment of nanoparticle cytotoxicity to cancer cells. Precise control over the storage of different types of molecules enables the optimization of nanoparticle properties for simultaneous drug delivery and imaging.
DNA-Gold Nanoparticles	Gold nanoparticles can be functionalized with DNA, creating drug carriers in gene therapy and drug delivery.	[157]	The chemotherapeutic agent cisplatin was chemically linked to pGEM-3Zf(−) plasmid DNA to produce a cisplatin-DNA complex. Gold nanoparticles, which bind electrostatically to pure DNA, could also be added to this complex. Dry films of pure plasmid DNA and DNA-cisplatin, DNA-gold nanoparticles, and DNA-cisplatin-gold nanoparticles complexes were bombarded by 60 keV electrons. The yields of single- and double-strand breaks were measured by electrophoresis as a function of exposure. From a comparison of such yields from the different types of films, we found that the binding of only one gold nanoparticle to a plasmid-cisplatin complex containing 3197 base pairs increases by a factor of 3 the efficiency of the chemotherapeutic agent cisplatin to produce double-strand breaks in irradiated DNA. Furthermore, adding two cisplatin molecules and one gold nanoparticle to DNA enhances radiation-induced Double-Strand Breaks (DSBs) by a factor of 7.5. A number of phenomena could contribute to this enormous enhancement, including the higher density of low-energy electrons and reactive species around the gold nanoparticles and the weakening of bonds adjacent to cisplatin in the DNA backbone. The addition of gold nanoparticles to cisplatin and other platinum agents may provide exciting avenues of research to improve cancer treatment by concurrent chemoradiation.
		[158]	Gold nanoparticles functionalized with DNA are among nanobiotechnology’s most commonly used reagents. DNA-functionalized AuNPs are very stable in the buffer (without PEG), and citrate-capped AuNPs are very stable in PEG. DNA-functionalized AuNPs are unstable in PEG and easily undergo aggregation.
SiRNA-Chitosan Nanoparticles	Chitosan nanoparticles with siRNA could potentially be used to treat Huntington’s disease (HD)	[159]	Nose-to-brain delivery of chitosan-based nanoparticles enriched with siRNA against Huntingtin (HTT) was studied in a transgenic YAC128 mouse model of Huntington’s Disease. A series of chitosan-based nanoparticle formulations were developed to encapsulate anti-HTT siRNA, protecting the payload from degradation en route to the target. Factors improving the production of effective nanocarriers for anti-HTT siRNA were identified and tested in a YAC128 mouse model of Huntington’s disease. Four nanocarrier formulations were identified as effective in lowering HTT mRNA expression by at least 50%. Intranasal administration of nanoparticles carrying siRNA presents a promising therapeutic alternative for the safe and effective reduction of mutant HTT expression.
ShRNA-Chitosan Nanoparticles	Nanostructured carriers for anticancer therapy applications	[160]	Short hairpin RNA (ShRNA) associated with methylenetetrahydrofolate dehydrogenase 1-like (MTHFD1L), delivered through CS-TPP-(shMTHFD1L-ALA) nanoparticles, effectively increased cytotoxicity against oral squamous cell carcinoma (OSCC) cells compared to other groups. Chitosan, serving as the carrier for CS-TPP-(shMTHFD1L-ALA) nanoparticles, demonstrated effective delivery of shRNA to cells, contributing to a more substantial proapoptotic and anticancer effect. CS-TPP-(shMTHFD1L-ALA) exhibited additional therapeutic effects both in vitro and in vivo, suggesting this platform’s potential application in treating oral squamous cell carcinoma. These results underscore the promising properties of chitosan as a carrier for shRNA, opening avenues for further research on this delivery system in the context.
MiRNA-Chitosan Nanoparticles	Nanostructured carriers for anticancer therapy applications	[161]	Chitosan demonstrates potential as a carrier for carbon dots, confirmed by the effective delivery of plasmid DNA (EGFP-N1) and miRNA-153 to cancer cells. Chitosan-based carbon dots (CS-CDs) exhibited non-cytotoxic behavior up to a concentration of 200 μg/mL, suggesting good cell tolerance. Combining chitosan with polyethyleneimine (PEI) in carbon dots (CP25-CDs) showed adequate plasmid DNA and miRNA protection from serum-induced degradation, confirming their stability. CP25-CDs demonstrated the ability to efficiently deliver miRNA-153 to cells, resulting in significant cell death in the examined cancer cell lines. CS-CDs and CP2-CDs (carbon dots with chitosan and PEI-2kDa) were well-tolerated by cells, highlighting the potential application of these nanocarriers in therapy without exhibiting toxicity. The CP25-CDs complex appears promising as an innovative vector for efficient miRNA delivery, suggesting its potential use in cancer therapy, particularly in treating oral squamous cell carcinoma.

**Table 4:** Examples of tested drug-chitosan nanocarriers and drug-chitosan nanocomposite combinations in various therapeutic therapies.

Chitosan-	Drug	Proven Results	Ref.
based drug carriers	Doxorubicin	Dual-scale Graphene Quantum Dots-Titania Nanoparticles (GTA), composed of glycolic acid, lactic acid, and all-trans retinoic acid double grafted onto N,N,N-trimethyl chitosan, were designed for the co-delivery of DOX and siRNA against Bcl-2. GTA nanoparticles exhibited selective toxicity to cancer cells (QGY-7703) at a 0.1 mg/mL concentration while maintaining low toxicity levels for normal cells. GTA/DOX/siRNA nanoparticles demonstrated high cellular uptake through receptor-mediated endocytosis with DOX in the hydrophobic core and siRNA on the hydrophilic shell. Due to the collaborative antitumor effects of DOX, siBcl-2, and GTA nanoparticles, GTA/DOX/siRNA nanoparticles showed superior in vitro and in vivo antitumor efficacy compared to other formulations. These results suggest the promising potential of GTA/DOX/siRNA nanoparticles as an effective strategy for delivering anticancer drugs.	[175]
		Chitosan-tripolyphosphate nanoparticles (CS-NPs) loaded with DOX were constructed, and their pH sensitivity allowed for membranolytic behavior in endosomal compartments. Modifying nanoparticles with PEG and poloxamer can potentially enhance the effectiveness of cancer treatments. DOX-loaded NPs exhibited higher cytotoxicity against tumor cells than the free drug, suggesting their potential anticancer efficacy. CS-NPs with DOX showed greater selectivity for tumor cells than non-tumor cells, indicating potential targeting to the tumor microenvironment. The ability of NPs to maintain hemocompatibility suggests their suitability for intravenous administration. Combining endosomal acidity with the potential endosomolytic capability of CS-NPs could increase the intracellular delivery of DOX and potentially enhance its anticancer efficacy.	[176]
	Paclitaxel	The anticancer drug was encapsulated within selenium nanoparticles modified with chitosan. The average size of developed nanoformulations was 170 nm. Nanoparticles showed sustained drug release ability for 72, which proceeded faster in slightly acidic conditions. Cytotoxicity of nanoparticles towards human cervical carcinoma cells was reported.	[177]
	Curcumin	Innovative T7 peptide-modified nanoparticles (T7-CMCS-BAPE, CBT) based on carboxymethyl chitosan were developed, capable of precisely binding to the transferrin receptor (TfR) on lung cancer cells. CBT nanoparticles enable precise drug release regulation based on pH and reactive oxygen species (ROS) levels. The drug-loading content of docetaxel (DTX) and curcumin (CUR) was approximately 7.82% and 6.48%, respectively, with the nanoparticles demonstrating good biocompatibility. T7-CMCS-BAPE-DTX/CUR complexes (CBT-DC) exhibited superior in vitro and in vivo anticancer effects compared to DTX monotherapy and other carriers loaded with DTX and CUR. CBT-DC demonstrated the ability to improve the immunosuppressive microenvironment, contributing to tumor growth inhibition. The findings provide a promising foundation for developing combined lung cancer therapy strategies.	[178]
		The study demonstrates the effectiveness of magnetic-guided targeting in delivering curcumin diethyl γ-aminobutyrate (CUR-2GE), a prodrug of CUR, previously synthesized to overcome unfavorable physicochemical properties of CUR, to breast cancer cells. Optimal conditions for preparing chitosan-coated iron oxide nanoparticles (Ch-IONPs) included using 4 mg of chitosan at pH 11 under a reaction temperature of 85 °C. Ch-IONPs were successfully loaded with CUR-2GE, achieving significant loading capacity (1.72%) and encapsulation efficiency (94.9%). CUR-2GE-loaded Ch-IONPs exhibited desirable characteristics: a particle size below 50 nm based on TEM images, superparamagnetic properties, a highly crystalline iron oxide core, stability, and a controlled release profile. In the presence of permanent magnets, CUR-2GE-loaded Ch-IONPs significantly increased cellular uptake and cytotoxicity against MDA-MB-231 cells compared to free CUR-2GE, indicating the potential of magnetic-field-assisted delivery for triple-negative breast cancer treatment.	[179]
based nanocomposites	Ibuprofen	A ternary nanogel based on chitosan-ibuprofen-gellan has been developed to control the transdermal delivery of ibuprofen. Contemporary nanoconjugates of ibuprofen and chitosan exhibited a significant reduction in the size of ibuprofen particles, along with thermal stability and an amorphous character. The nanogels demonstrated significant elastic and pseudoplastic properties, with the maximum swelling capacity observed at 6.55 mM chitosan. Chitosan improved skin penetration, permeability, and the rate of transdermal release of ibuprofen, attributed to ion interactions and chitosan concentration.	[180]
	Methotrexate	MTX-PEG-CS-IONPs-Cy5.5 nanocomposites were developed by combining chitosan-decorated iron oxide nanoparticles (CS-IONPs) with polyethylene glycolated methotrexate (MTX-PEG) and the fluorescent dye cyanine (Cy5.5). MTX-PEG-CS-IONPs-Cy5.5 demonstrated the ability to synergistically combine early-phase selective tumor targeting with a late-phase cancer-killing effect. The nanocomposites’ dual imaging capabilities (magnetic resonance and fluorescence) were confirmed for monitoring the treatment process. MTX-PEG, acting as a PEGylated anticancer prodrug, served as a tumor-cell-specific targeting ligand, enabling targeted cancer treatment. The nanocomposites effectively delivered MTX to cancer cells with overexpressed folate receptors (FA), improving anticancer activity and reducing side effects. Utilizing folate receptor-mediated endocytosis and pH/intracellular protease-mediated hydrolysis of peptide bonds allowed precise drug release regulation under specific conditions.	[181]
	Insulin	Nanocomposites were obtained through alginate gelation and an electrostatic interaction process of polyelectrolytes. Chitosan significantly influenced the final size of the nanocomposites, with an optimal content resulting in a hydrodynamic size of 400–600 nm. Improved stability of the nanocomposites was confirmed post-synthesis using LUMiSizer. Stability over time and under varying ionic strength and pH conditions was assessed through dynamic light scattering. The rounded shapes of the nanocomposites were verified using scanning electron microscopy. Upon loading with insulin, the nanocomposites exhibited complete drug release under physiologically simulated conditions.	[182]
